# Relationship and mental health outcomes after childbirth among women with endometriosis: An 8-year follow-up study

**DOI:** 10.1177/17455057261471816

**Published:** 2026-07-22

**Authors:** Marius Johansen, Tone Kristin Omsland, Katariina Laine, Maria Christine Magnus

**Affiliations:** 1 Department of Community Medicine and Global Health, Institute of Health and Society, 6305 University of Oslo, Oslo, Norway; 2 Norwegian Research Centre for Women’s Health, Oslo University Hospital, Oslo, Norway; 3 Institute of Clinical Medicine, Faculty of Medicine, 6305 University of Oslo, Oslo, Norway; 4 Centre for Fertility and Health, Norwegian Institute of Public Health, Oslo, Norway

**Keywords:** endometriosis, relationship dissolution, relationship satisfaction, anxiety, depression, the Norwegian Mother, Father and Child Cohort Study, the Medical Birth Registry of Norway

## Abstract

**Background:**

Transitioning to parenthood is stressful for relationships. Women with endometriosis face several health challenges, which may further strain their relationships when they become parents. However, it remains uncertain whether this transition compromises their relationships beyond what is observed in the general population.

**Objectives:**

To compare the risk of relationship dissolution, relationship satisfaction, anxiety, and depression between women with and without endometriosis.

**Design:**

Longitudinal population-based cohort study including 99,531 singleton pregnancies to 84,642 women.

**Methods:**

1,476 pregnancies to women with self-reported endometriosis were compared to 98,064 pregnancies in women without endometriosis, using data from the Norwegian Mother, Father and Child Cohort Study (1999-2017). Women were followed up to 8 years postpartum. Adjusted risk ratios (aRR) with 95 % confidence intervals (CI) were calculated by multivariable log-binomial regression, adjusting for age, education and income.

**Results:**

We observed increasing trends of mothers not living with the biological father (12% at 8 years postpartum) and of relationship dissatisfaction (4.7% at 5 years postpartum), without significant differences in the risk of relationship dissolution (10% vs. 12%) or dissatisfaction (4.8% vs. 4.7%) between women with and without endometriosis. However, women with endometriosis had significantly higher risks of anxiety (aRR: 1.30, 95% CI: 1.06-1.58) and depression (aRR: 1.32, 95% CI: 1.16-1.50) at 8 years postpartum.

**Conclusion:**

Mothers with endometriosis did not exhibit increased relationship dissolution or dissatisfaction compared to those without, despite experiencing a greater mental health burden. While these findings are encouraging, they should not trivialize the potential impact of endometriosis on quality of life.

## Introduction

Endometriosis is a chronic gynaecological condition affecting approximately 10% of women, with painful menstruations as the cardinal symptom.^1^ Over time, it can progress to chronic abdominal pain, often accompanied by fatigue, dyspareunia, and decreased fertility.^2,3^ Beyond physical symptoms, endometriosis significantly impacts mental health, resulting in higher rates of anxiety and depression and impaired quality of life.^4–6^ Many women with endometriosis encounter disbelief, stigma, and lack of support from healthcare providers and social circles, including partners and family, which, along with limited awareness of the condition, often leads to significant diagnostic delays and heightened emotional distress.^6–9^

Endometriosis is frequently associated with relationship strain, mainly due to dyspareunia and sexual dysfunction.^10–14^ Partners may also face psychological and practical burdens.^15,16^ Although chronic illness in either partner seems to increase the risk of separation or divorce,^17^ no analytical epidemiological studies have quantified this risk specifically for endometriosis. Existing evidence is suggestive but limited by small, selected samples and cross-sectional, retrospective, or qualitative designs.^14,18,19^ Findings on relationship satisfaction are sparse and mixed.^20,21^

Transitioning to parenthood presents challenges such as division of labour, differing parenting styles, and restricted freedom, often leading to lower marital satisfaction compared to nonparents.^22,23^ We were particularly interested in whether this transition differs for women with endometriosis compared to those without. Notably, no studies have explicitly explored the association between endometriosis and relationship satisfaction in couples with children.

Although growing evidence links endometriosis to increased anxiety and depression,^4,5,24–26^ comparisons across settings are limited by the lack of universally accepted, validated measurement tools.^5^ We identified no studies focused on longitudinal mental health outcomes in new mothers with endometriosis, apart from three cohort studies that examined postpartum depression risk.^27–29^

Therefore, our study aimed to compare the risk of relationship dissolution and measures of relationship satisfaction between women with and without endometriosis up to 8 years postpartum. A secondary aim was to explore whether mothers with endometriosis were more likely to experience anxiety and depression during this period compared to women without the condition.

## Materials and methods

### Study population

The Norwegian Mother, Father, and Child Cohort Study (MoBa) is a population-based pregnancy cohort initiated by the Norwegian Institute of Public Health (NIPH). MoBa recruited over 95,000 women with more than 112,000 pregnancies, at approximately 15 weeks of gestation, between 1999 and 2008. The age range was 15-48 years. Some women participated with multiple pregnancies. The participation rate was 41%.^30^

A 1997 pilot preceded the cohort launch.^31^ After initial returns, questionnaires were evaluated with multi-institutional input and revised accordingly.^31^ For many topics/outcomes, existing validated instruments or short forms of validated scales were used (e.g., the Hopkins Symptom Checklist^32^). For other topics/outcomes, instruments were developed specifically for MoBa (e.g., the Relationship Satisfaction Scale^33^).

Our study utilized self-reported data collected through the recruitment questionnaire (during pregnancy) and the mothers’ follow-up questionnaires at 6 months, 18 months, and at 3, 5, and 8 years postpartum. All MoBa questionnaires are available online.^34^ The MoBa data were supplemented with linkage to birth records from the Medical Birth Registry of Norway (MBRN) using unique identification numbers.

**Figure 1.**
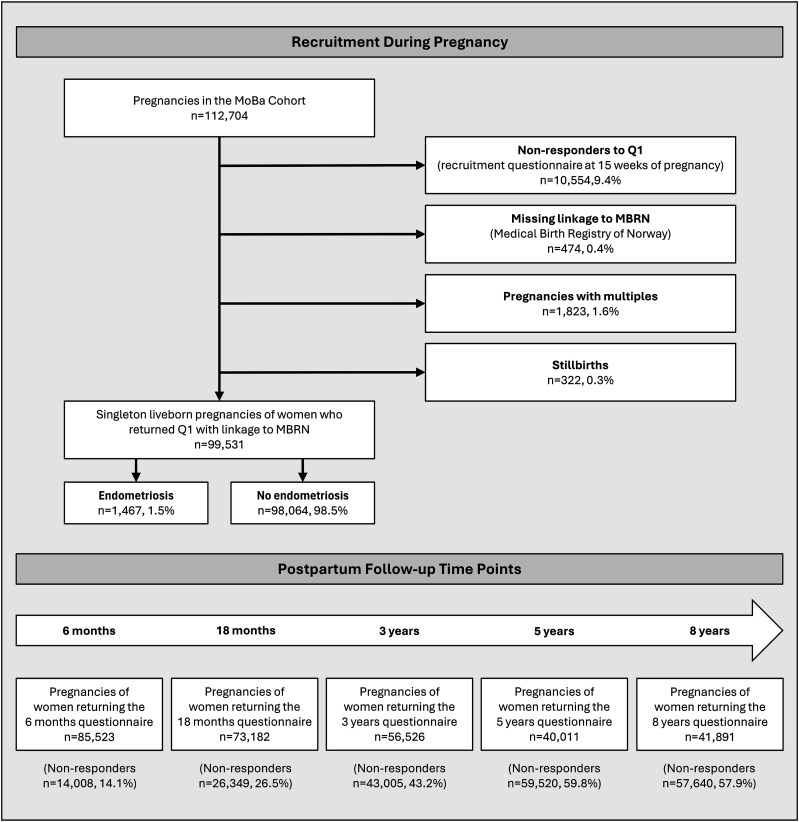
Illustration of study population.

We included all the pregnancies in the MoBa cohort (n=112,704). Exclusion criteria were non-responders to the recruitment questionnaire, those missing linkage to MBRN, pregnancies with multiples, and stillbirths (Figure 1).

Reporting followed the Strengthening the Reporting of Observational Studies in Epidemiology (STROBE) guidelines^35^ (Supplementary Material 1).

### Endometriosis

Endometriosis was self-reported in the recruitment questionnaire where participants listed prior diagnoses. Diagnostic method and age at diagnosis were not collected, and the self-reported diagnosis could not be validated against clinical records within the scope of this project.

During the time span of our cohort (1999-2008), we had no reliable sources beyond MoBa to ascertain endometriosis diagnoses. Although the Norwegian Patient Registry (NPR) was established in 2008, and contains ICD-10 codes retrospectively to 1997, linkage was not feasible because NPR first became person-identifiable in 2008.^36^ In theory, augmenting MoBa with NPR or other registry data could have improved exposure ascertainment, but this was not possible for our cohort.

### Relationship outcomes

To assess relationship dissolution from the biological father, we restricted the analyses to participants who reported being in a relationship (married or cohabiting) at the time of recruitment. However, it should be noted that the initial data did not specify whether the reported relationship was with the child’s biological father. We collected follow-up responses to the question, ‘Do you live with your child’s father?’ Participants could answer ‘Yes’ or ‘No’. This question was included in all but the 6-month questionnaires.

Relationship satisfaction was measured among all women with a partner, regardless of whether the partner was the child’s father, using the Relationship Satisfaction Scale (RSS).^33^ The RSS is a 10-item questionnaire developed for MoBa, measuring marital satisfaction and relationship quality, with responses rated on a 6-point Likert scale, ranging from ‘strongly disagree’ to ‘strongly agree’. Confirmatory factor analyses support a unidimensional structure with high loadings and good fit. The RSS correlates strongly with the Quality of Marriage Index (r≈.92),^37^ and shows predictive validity for future break-up/divorce and life satisfaction.^33,38^

While the full 10-item RSS was used in the 6- and 18-months questionnaires, a shorter 5-item RSS version (correlating r≈.97 with the full scale) was used in the 3- and 5-years questionnaires.^33^ The RSS was not included in the 8-years questionnaire. For comparability, we used the shorter 5-item RSS version at all time points where participants answered the RSS. A cutoff score of ≥4.0 from the mean was used to identify those in a dissatisfactory relationship.^33^

The 5- and 10-item RSS questionnaires are presented in the supplementary material.

### Mental health outcomes

Anxiety and depression were assessed among all participating women, regardless of relationship status, using the Hopkins Symptoms Checklist.^39^ The 8-item version (SCL-8) included in all questionnaires consists of four items related to anxiety symptoms and four items related to depression symptoms.^32^ Responses for each item are rated on a 4-point scale from 1 (‘not at all’), to 4 (‘extremely’). The average score (ranging from 1.00 to 4.00) was employed as a measure of distress. Our analysis used a cutoff score of ≥1.75 on the SCL-8 anxiety and depression subscale means to indicate clinically significant symptoms, consistent with prior HSCL validation studies,^40^ and previous SCL-8 MoBa studies.^41^

The accuracy of SCL-8 is supported by the SCL-25, a more comprehensive 25-item version, which demonstrated a high degree of agreement (86.7%) between patients’ self-reported symptoms and physician assessments.^42^ Research indicates high correlation and reasonably strong reliability and measurement precision between the scales (SCL-8 and SCL-25) for both anxiety and depression scores, suggesting that the shorter SCL-8 is also a reliable tool for assessing these mental health conditions.^32^

The SCL-8 scale is presented in the supplementary material for further reference.

### Covariates

Information on maternal age at delivery and parity was extracted from the MBRN. From the MoBa recruitment questionnaires, we further obtained self-reported data on educational attainment (categorized as less than high school, high school, up to 4 years of college, and >4 years of college), and annual income (categorized as low [<200,000 NOK (Norwegian krone)], medium [200,000-399,999 NOK], and high [>400,000 NOK]). Missing data were grouped into separate categories.

Maternal age was identified as the key confounder affecting both endometriosis (exposure) and relationship- and mental outcomes.^43^ We also considered education and income as proxies for the women’s socioeconomic status during their upbringing. We deliberately avoided adjusting for factors like parity and a history of mental problems (including antenatal- and postpartum depression), as we considered these variables to be intermediate factors.^1,2,44,45^

### Statistical analyses

We employed log-binomial regression to compare the risk of four relationship and mental outcomes (relationship dissolution, relationship dissatisfaction, and symptoms of anxiety and depression) between women with and without endometriosis. We calculated crude and adjusted relative risks (RR and aRR) with 95% confidence intervals (CI). Adjustments were made in two steps: first for maternal age (continuous), then for maternal age and socioeconomic status (categorical, reflecting education and income levels, as presented in Table 1).

**Table 1.** Background characteristics according to endometriosis status among 99,531 singleton pregnancies in the Norwegian Mother, Father and Child cohort study (1999-2008).

​	Endometriosis n=1,467 (1.5 %)		No reported endometriosis n=98,064 (98.5 %)	
**Maternal age (SD) at delivery**				
Age at birth, mean year	32.87	(4.16)	30.63	(4.59)
*Missing*	*0*	​	*0*	​
**Parity at index pregnancy**				
Nulliparous	714	(48.67 %)	43,859	(44.72 %)
Multiparous	753	(51.33 %)	54,205	(55.28 %)
*Missing*	*0*	​	*0*	​
**Relational status at recruitment**				
Married or cohabiting	1,417	(97.52 %)	94,274	(96.63 %)
Other^a^	36	(2.48 %)	3,291	(3.37 %)
*Missing*	*14*	​	*499*	​
**Level of completed education at recruitment**				
Less than high school	108	(7.40 %)	7,897	(8.09 %)
High school	397	(27.21 %)	28,901	(29.62 %)
Up to 4 years of college	589	(40.37 %)	39,103	(40.08 %)
>4 years of college	365	(25.02 %)	21,661	(22.20 %)
*Missing*	*8*	​	*502*	​
**Annual income at recruitment**				
Low (0-199.999 NOK)	298	(20.85 %)	28,403	(30.04 %)
Medium (200.000-399.999 NOK)	910	(63.68 %)	55,249	(58.44 %)
High (>400.000 NOK)	221	(15.47 %)	10,894	(11.52 %)
*Missing*	*38*	​	*3,518*	​

Abbreviations: NOK: the Norwegian krone, currency of Norway.

^a^Includes divorced/separated, widowed, single, or ‘other’.

We conducted available-case analyses by outcome and time point: participants with missing outcome data at a given wave were excluded from that analysis but could contribute to others and to later waves. We did not perform imputation or complete-case analysis. For covariates with small amounts of missing data (education: 0.5%; income: 3.6%), we included a ‘missing’ category to retain participants and adjust for these confounders (proxies for socioeconomic status). To account for dependencies related to multiple pregnancies in the same women, we adjusted standard errors using robust cluster variance. We also assessed effect modification by parity via interaction terms.

Stata version 18 (StataCorp, College station, TX, USA) was used for the analyses.

### Sample size

This study is a secondary retrospective analysis of prospectively collected MoBa cohort data. Consequently, the sample size was determined by cohort enrolment and data availability. No a priori power calculation was performed. Analyses used the full eligible sample to maximize statistical precision, and we present 95% CIs for all key estimates.

We restricted the analysis to liveborn singleton pregnancies in women with information from the recruitment questionnaires completed at 18 gestational weeks. We excluded 474 pregnancies with missing linkage to MBRN, resulting in a study population of 99,531 pregnancies (Figure 1). Endometriosis was self-reported by women in 1,476 pregnancies (1.5%), with the remaining 98,064 pregnancies classified as unexposed. The corresponding sample sizes for each follow-up questionnaire (6 months, 18 months, and 3, 5, and 8 years postpartum) are presented in Figure 1.

## Results

Women with endometriosis were generally older (maternal age at delivery: 32.9 vs. 30.6 years), more often nulliparous at the index pregnancy (49% vs. 45%, assessed at approximately 15 gestational weeks), had a higher level of completed education (25% with >4 years of completed education vs. 22%), and a higher income (15% with high annual income vs. 12%) than women without endometriosis (Table 1).

**Figure 2.**
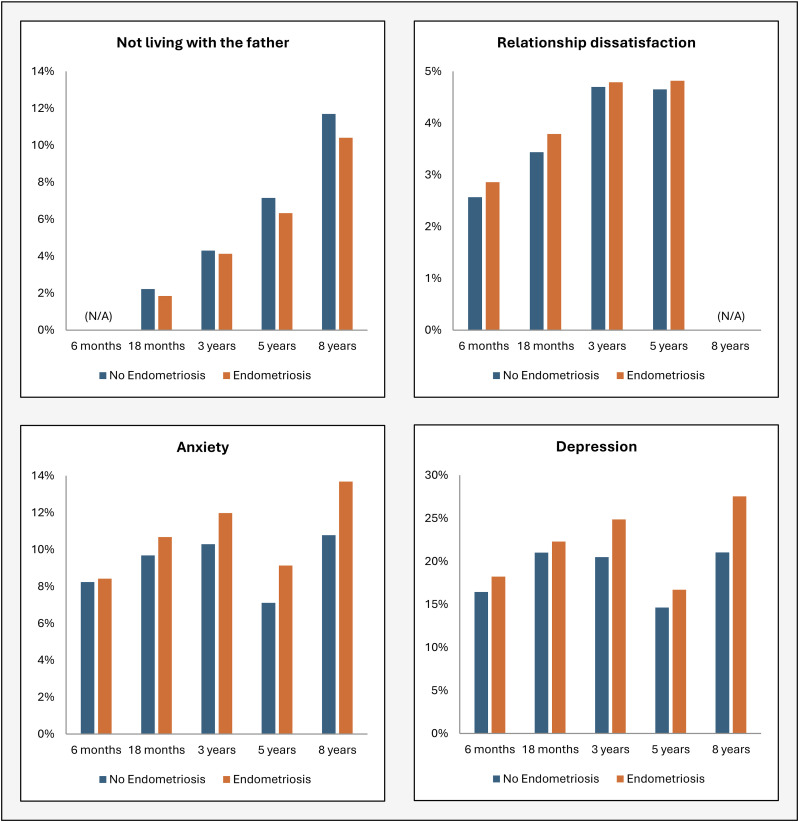
Frequencies of relational and mental health outcomes.

The prevalence of anxiety and depression showed increasing trends from 6 months to 8 years postpartum in the overall population (Figure 2). Women with endometriosis consistently exhibited borderline or significantly higher risks for both mental health outcomes compared to those without endometriosis, with the greatest difference observed at 8 years postpartum: anxiety prevalence was 13.7% versus 10.8% with an aRR of 1.30 (95% CI: 1.06-1.58), and depression prevalence was 27.5% versus 21.0% with an aRR of 1.32 (95% CI: 1.16-1.50) (Supplementary Table 1).

Response rates were reasonable high at recruitment (>90%) but declined over time, reaching their lowest at 8 years (∼37%). Outcome-specific missingness ranged from ∼1% (relationship dissolution at 18 months) to ∼10% (depression at 6 months). Analysis-specific sample sizes are reported in Tables 1 and 2 and Supplementary Table 1.

**Table 2.** Relative risk (RR) of relationship dissolution in women with endometriosis (pregnancies with no reported endometriosis as reference) among 95,691 liveborn singleton pregnancies (of women who were married or cohabiting at the time of recruitment) in the Norwegian Mother, Father and Child cohort study (1999-2008), with follow-up at 18 months, and at 3, 5, and 8 years postpartum.

​	Endometriosis n cases/n total (percentage)		No reported endometriosis n cases/n total (percentage)		Unadjusted RR		Adjusted RR^a^		Adjusted RR^b^	
**Relationship dissolution**										
18 months postpartum	20/1,081	(1.85 %)	1,531/68,959	(2.22 %)	0.89	(0.70-1.13)	1.09	(0.71-1.69)	1.01	(0.66-1.57)
3 years postpartum	36/871	(4.13 %)	2,272/52,855	(4.30 %)	0.96	(0.69-1.34)	1.22	(0.88-1.69)	1.14	(0.82-1.58)
5 years postpartum	39/616	(6.33 %)	2,713/37,925	(7.15 %)	0.89	(0.64-1.22)	1.04	(0.75-1.43)	0.98	(0.71-1.35)
8 years postpartum	68/653	(10.41 %)	4,612/39,435	(11.70 %)	0.87	(0.69-1.10)	0.99	(0.79-1.26)	0.96	(0.76-1.22)

^a^adjusted for maternal age at birth.

^b^adjusted for maternal age at birth and socioeconomic status (maternal education and income).

### Relationship outcomes

At recruitment, 96.6% of the women were married or cohabiting, showing no significant difference between women with and without endometriosis (97.5% vs. 96.6%). Women registered as divorced/separated, widowed, single, or other were categorized as not being in a relationship (Table 1).

Among mothers who were in a relationship at the time of recruitment, 3.8% were no longer living with the biological father of their child by 18 months postpartum. This increased over time, reaching 11.7% at 8 years postpartum in the total study population (Figure 2). However, there was no significant difference between women with and without endometriosis. The prevalence of relationship dissolution was 10.4% vs. 11.7% at 8 years postpartum, with an aRR of 0.96 (95% CI: 0.76-1.22) (Table 2).

Similarly, dissatisfaction in relationships increased over time from 2.6% at 6 months postpartum to 4.7% at 5 years postpartum in the overall population (Figure 2). We found no difference in relationship dissatisfaction between women with and without endometriosis. At 5 years postpartum the prevalence of relationship dissatisfaction was 4.8% vs. 4.7% with an aRR of 0.88 (95% CI: 0.61-1.28) (Table 3).

**Table 3.** Relative risk (RR) of relationship dissatisfaction in women with endometriosis (pregnancies with no reported endometriosis as reference) among 99,531 liveborn singleton pregnancies in the Norwegian Mother, Father and Child cohort study (1999-2008), with follow-up at 6 months, 18 months, and 3 and 5 years postpartum.

​	Endometriosis n cases/n total (percentage)		No reported endometriosis n cases/n total (percentage)		Unadjusted RR			Adjusted RR^a^		Adjusted RR^b^	
**Relationship dissatisfaction**											
6 months postpartum	35/1,222	(2.86 %)	2,044/79,450	(2.57 %)	1.11	(0.79-1.56)	1.01		(0.72-1.42)	1.00	(0.74-1.36)
18 months postpartum	40/1,056	(3.79 %)	2,336/67,965	(3.44 %)	1.10	(0.81-1.50)	1.01		(0.75-1.38)	1.00	(0.73-1.37)
3 years postpartum	41/856	(4.79 %)	2,445/52,012	(4.70 %)	1.02	(0.74-1.40)	0.92		(0.67-1.26)	0.91	(0.66-1.24)
5 years postpartum	29/602	(4.82 %)	1,714/36,840	(4.65 %)	1.04	(0.71-1.50)	0.89		(0.61-1.29)	0.88	(0.61-1.28)

^a^adjusted for maternal age at birth.

^b^adjusted for maternal age at birth, and socioeconomic status (maternal education and income).

### Interaction

We found no evidence of effect modification by parity (interaction p-values >0.10).

## Discussion

In this large Norwegian cohort study, following mothers for up to 8 years postpartum, we observed rising prevalences of relationship dissolution from the biological father and relationship dissatisfaction over time. Anxiety and depression rates were higher among women with endometriosis. Nevertheless, despite the increased mental health burden, there were no significant differences in relationship dissolution or relationship satisfaction between women with and without endometriosis. To our knowledge, this is the first follow-up study evaluating relationship satisfaction among women with endometriosis at multiple time points, and the first to assess relationship dissolution among these women. Additionally, it is the first to explore relationship and mental health outcomes among new mothers with the condition.

### Comparison with previous studies

The risk of relationship dissolution is higher among couples where one partner has a chronic disease, estimated to a 40% increase in a 2020 Australian study.^17^ However, we did not identify any epidemiological studies that have estimated the association between endometriosis and separation or divorce. Limited, suggestive evidence comes from a 2013 multinational cross-sectional survey,^18^ a 2007 UK qualitative study,^14^ and a 2009 Norwegian retrospective follow-up study,^19^ where participants often perceived endometriosis as contributing to relationship strain or breakup. These studies lacked control groups, had small or selected samples, and used designs not intended to quantify such risks.

We identified two studies that compared partnership satisfaction between women with and without endometriosis. A 2024 study conducted across Switzerland, Germany, and Austria, examined partnership quality (the German 30-item Partnerschaftsfragebogen, PFB), conflict areas (the 23-item Problem List, PL), and thoughts of separation among 381 women with, and 381 without endometriosis, in addition to their partners.^20^ Most participants in this study did not have children. Similar to our findings, no significant difference was observed in partnership quality ratings between women with and without endometriosis and their partners. However, women with endometriosis reported separation thoughts more frequently and experienced more partnership-related conflicts than those without the condition. A 2022 Spanish study compared 55 women with deep infiltrating endometriosis to 60 women without endometriosis, along with their partners, assessing quality of sex-life (the 18-item Sexual Quality of Life-Female, SQOL-F) and relational dyadic adjustment (the 32-item Dyadic Adjustment Scale, DAS). This study reported a significant decline in the quality of sex life, and, contrary to our results and those of the Swiss/German/Austrian study, an overall increase in relationship problems, particularly impairment in relationship satisfaction (a DAS subscale), among the endometriosis group.^21^ Notably, none of these, nor any other identified studies, have explored relationship satisfaction within a longitudinal or follow-up design framework. Furthermore, we found no research specifically addressing relationship aspects following motherhood for women affected by endometriosis.

Many studies report higher levels of anxiety and depression among women with endometriosis,^4,5,24,46^ though prevalence estimates vary widely, likely due to differences in study populations and heterogeneity in case identification.^5,24^ The increased mental health burden is attributed to pain,^4,5,46,47^ as well as impaired sleep and fatigue,^4^ sexual dysfunction,^4,48,49^ lower self-esteem and emotional self-efficacy,^4,50^ stigma,^51^ side effects of hormonal therapy and distress related to surgery,^5,52^ and the cumulative stress of living with a painful chronic disease.^52^

We have previously reported an increased risk of postpartum depression among women with endometriosis in the MoBa cohort.^29^ Two other cohort studies have also examined postpartum depression: a 2017 Japanese cohort^27^ reported increased risk among women with endometriosis, whereas a 2025 South African cohort found no increase.^28^ To our knowledge, no studies have evaluated the long-term risks of anxiety and depression in mothers with endometriosis.

### Potential explanatory mechanisms

Just as with other chronic diseases, women with endometriosis face substantial psychosocial burdens, including challenges that can affect intimate relationships.^6,53^ Many report feeling disbelieved by clinicians, partners, family, and in social settings, leading to stigmatization, limited support, loneliness, and isolation.^13,53,54^ Increased healthcare use^55^ and impaired work or career attainment^53,56–58^ can adversely affect personal and family finances.^15,59^ Endometriosis has been linked to higher rates of alcohol and drug dependence,^52^ and reliance on strong analgesics may increase the risk of opioid dependence.^60^ In addition to anxiety and depression, associations have been reported with bipolar disorder,^26^ psychosis,^52^ personality disorders,^26^ eating disorders,^52^ and elevated risk of self-harm and suicide.^61^

Challenges within couple relationships are commonly related to dyspareunia and sexual dysfunction.^10–14^ Difficulties conceiving and infertility can further strain relationships.^53^ Partners may also experience psychological and practical burdens, including stress, reduced sexual satisfaction, and caregiving strain.^15,16^

In light of this, and contrary to our expectations, it was surprising to find no differences in relationship dissolution or dissatisfaction between women with and without endometriosis. This was especially unexpected given our findings that, at 8 years postpartum, women with endometriosis faced significantly higher risks of anxiety (30%) and depression (32%), issues that can profoundly affect quality of life and relationships.^24,62^

The results suggest relatively stable relationship functioning in women with endometriosis who become pregnant. However, caution is warranted in inferring that this pattern reflects distinct relational protective mechanisms that can mitigate the negative impacts of endometriosis on couple relationships. Nevertheless, some qualitative studies suggest a possible supportive effect, indicating that couples may interact in ways that foster emotional intimacy, understanding and empathy, and that these processes might help to mitigate relationship dissatisfaction.^15,16,63,64^ Couples coping with endometriosis have reported deepened communication and closeness through mutual support and acceptance.^15,16,64^ Furthermore, engaging in open communication and focusing on abilities rather than limitations has been linked to greater relational closeness and resilience.^65^

Research on other chronic diseases underscores the benefits of dyadic coping, where couples communicate, support one another, and manage stress collectively. A 2021 systematic review of 49 studies, including one on endometriosis, identified positive dynamics such as stress communication, humour, mutual acceptance of illness, shared stress management, and viewing chronic conditions as shared tasks.^66^ These factors fostered positive relationship dynamics, enhance coping, intimacy, emotion regulation, and supported recovery from illness.^66^

Our findings of increasing trends in relationship dissolution and dissatisfaction, anxiety, and depression over time reflect well-known challenges of lower marital satisfaction among parents compared to non-parents.^22,23^ Transitioning to parenthood may shift focus away from individual health issues, potentially reducing attention on endometriosis-related dissatisfaction or overshadowing its impacts. This shift might explain the lack of observed relationship differences between women with and without endometriosis.

### Implications

Our findings of no increased relationship dissolution or dissatisfaction rates among mothers with endometriosis may suggest resilience among affected couples and could indicate that partner relationships constitute a supportive arena for affected women. However, the observed increase in anxiety and depression among these women highlights the need for targeted mental health support. The findings underscore the importance of distinguishing between individual psychological burden and relational outcomes. Healthcare professionals should proactively identify and address mental health issues associated with endometriosis, integrating psychoeducation and mental health screening into follow-up care, while recognizing the couple’s potential capacity as a coping resource. Learning from those with successful coping strategies and emphasising strengths and adaptive behaviours may further reinforce positive relationship dynamics. More targeted interventions, like structured couple-based programs and clinician training to identify relational strain, warrant evaluation in future studies.

Nonetheless, involving both partners in healthcare interventions, as they share the impact of the illness, improves relationship dynamics and disease outcomes for those with chronic diseases like endometriosis.^66^ Yet partners are rarely engaged by healthcare professionals.^15,67^ Inclusion and training in partner validation and empathy can enhance intimacy and emotional regulation in relationships, vital for adapting to chronic illness challenges.^68^

Finally, this study highlights key research gaps regarding relational and mental health, underscoring the need for well-designed longitudinal studies with comparison groups that track couples before and during the transition to parenthood, as well as those experiencing infertility or remaining childfree. In addition, factors not examined here (e.g., symptoms, sexual functioning, fertility and pregnancy factors, psychosocial variables, relationship dynamics, healthcare trajectory, biomarkers) should be evaluated as potential mediators and effect modifiers to clarify their roles in the association between endometriosis and relationship satisfaction.

### Limitations

The primary strengths of our study include its large size MoBa cohort (>90,000 singleton pregnancies) and extended follow-up period, enabling trajectory analyses and providing robust statistical power and generalizability. This is the largest study to date comparing relationship satisfaction among women with endometriosis to a control group, and the first to assess relationship dissolution rates and mental health outcomes through a longitudinal analysis across multiple time points, as well as the first to explore these outcomes following birth. Our broad recruitment from the general population contrasts with previous research that often focused on specific contexts like tertiary healthcare settings or patient advocacy groups.

However, because our analyses focus on women who achieved pregnancy and delivered, our findings are not generalizable to women with endometriosis who do not conceive or remain childless and likely underrepresent those with the most severe disease and persistent infertility. Psychosocial characteristics and disease severity may differ between women who become mothers and those who do not, potentially influencing relationship and mental health outcomes. Women who remain childless may experience a higher burden of pain and symptoms, as well as infertility-related stressors, which could lead to different or more adverse relationship and mental health trajectories. Accordingly, our findings should be interpreted as applying to women with endometriosis who achieved pregnancy and delivered. Moreover, comparability with other countries depends on diagnostic practices, referral pathways, ART availability and funding, and social welfare supports. These system-level factors can modify observed associations, particularly for mental health and relationship outcomes that are sensitive to cultural norms and access to services.

MoBa’s 41% participation rate, skewed toward higher socioeconomic status,^69^ may also affect external validity. Although we have no indication that research participation differed by endometriosis status per se, socioeconomic differences in diagnosis and access to care could influence who is identified as exposed and who enters the analytic cohort, potentially affecting observed associations.

Reliance on self-reported diagnoses prevented verification of disease stage and symptom severity. Women with endometriosis likely represent a heterogeneous group with respect to lesion extent, pain burden, and symptom severity. These clinical features may influence both relationship dynamics and mental health, and we were unable to examine whether associations differed by disease severity or to assess potential dose-response patterns. As a result, our estimates should be interpreted as average effects among women with self-reported endometriosis in this pregnancy cohort, and they may underestimate or mask stronger associations in women with more severe or highly symptomatic disease. Because the 1999-2008 cohort depended on self-reported endometriosis and registry validation was infeasible, as diagnostic codes were unavailable from relevant registries during this period, exposure misclassification cannot be ruled out. In the absence of comprehensive diagnostic, laparoscopic, or biopsy registries, self-report is often the only feasible approach in large epidemiologic cohorts. Although external validation was limited, several recent studies suggest that self-reported endometriosis has acceptable validity for epidemiological research,^70–73^ demonstrating substantial agreement, high sensitivity and specificity, and high accuracy relative to clinical records. Moreover, we consider it unlikely that in the early 2000s, when awareness and understanding of endometriosis were limited, many women would report endometriosis without a prior medical diagnosis. Consistent with prior literature, women in MoBa who self-reported endometriosis showed expected patterns, including a markedly higher frequency of infertility (47% vs 11%) and substantially higher use of ART (22% vs 2%),^29^ aligning with known associations with infertility^74^ and ART reliance.^75^ Such patterns support the credibility of self-reported exposure.

The observed prevalence of self-reported endometriosis in our cohort (1.5%) is lower than current estimates among women in general (∼10% when including undiagnosed cases^1,2,44^), but reasonably close to the 2% reported in a 1997 Norwegian study.^76^ Several features of our design and setting can explain a lower observed prevalence. First, because our sample includes only pregnancies, women with endometriosis-related infertility are underrepresented, which likely lowers the observed prevalence relative to the source population. Second, diagnostic delays and underdiagnosis increase the likelihood of underreporting at the time of the recruitment questionnaire.^1,2^ On the other hand, the cohort overrepresents highly educated women, who may be more likely to pursue infertility treatment, increasing diagnostic work-up and detection, and this could bias prevalence upward.

Another limitation is that the MoBa questionnaires did not measure cohabitation at 6 months and RSS at 8 years, which reduces temporal coverage for early postpartum and later childhood follow-ups. Follow-up participation also declined progressively across the 8 years postpartum, leading to substantial loss to follow-up. Outcome-specific missingness varied (e.g., approximately 1.0% for relationship dissolution at 18 months and 10.1% for depression at 6 months), and later-wave responders tended to be older, more highly educated, less likely to smoke, and had lower BMI,^77^ consistent with selective attrition. If women with poorer mental health, greater relationship strain, or more severe disease were less likely to continue participating, our estimates, particularly at later follow-ups, may be biased toward healthier, more stable profiles. Thus, the long-term mental health and relationship findings, especially at 8 years postpartum, should be interpreted with caution.

In this cohort, we did not systematically monitor drop-out patterns or compare characteristics of responders and non-responders at each wave, nor did we apply methods such as inverse probability weighting or multiple imputation for longitudinal data to address differential attrition. For socioeconomic status, we used a ‘missing indicator’ category for education and income to avoid loss of power. While this approach can introduce bias if missingness is related to exposure or outcomes, missingness was similar across exposure groups, suggesting limited risk of differential bias. Nonetheless, these features further underscore the possibility of selection bias due to loss to follow-up.

No a priori power calculations were conducted, as the sample size was determined by cohort enrolment and data availability, and post hoc power analyses were avoided because they are considered non-informative and conceptually inappropriate for interpreting completed studies.^78,79^ Moreover, despite efforts to control for confounding factors, residual and potential unmeasured confounding (like relationship quality prior to pregnancy, severity of endometriosis, or access to psychosocial support) may have influenced the observed associations.

Including fathers’ perspectives could have offered deeper insights. Additionally, relationship satisfaction measures did not specifically assess ties with the biological father, as relationship status at recruitment could theoretically involve another partner. However, this is unlikely to have significantly influenced the results, as our primary interest was in the overall impact on relationship dynamics.

## Conclusions

In this extensive Norwegian cohort study, mothers with endometriosis exhibited no significant differences in relationship dissolution or dissatisfaction compared to those without the condition, despite significantly higher levels of anxiety and depression. While these results are promising, they should not trivialize the burden of symptoms and potential profound impact of endometriosis on quality of life. We recommend including partners in the treatment and follow-up of endometriosis and encourage further comparative studies to explore relationship dynamics, including among women without children.

## Supplemental material

Supplemental material - Relationship and mental health outcomes after childbirth among women with endometriosis: An 8-year follow-up studyRelationship and mental health outcomes after childbirth among women with endometriosis: An 8-year follow-up study Supplemental material for Relationship and mental health outcomes after childbirth among women with endometriosis: An 8-year follow-up studyRelationship and mental health outcomes after childbirth among women with endometriosis: An 8-year follow-up study by Marius Johansen, Tone Kristin Omsland, Katariina Laine, and Maria Christine Magnus in Women’s Health.

Supplemental material - Relationship and mental health outcomes after childbirth among women with endometriosis: An 8-year follow-up studyRelationship and mental health outcomes after childbirth among women with endometriosis: An 8-year follow-up study Supplemental material for Relationship and mental health outcomes after childbirth among women with endometriosis: An 8-year follow-up studyRelationship and mental health outcomes after childbirth among women with endometriosis: An 8-year follow-up study by Marius Johansen, Tone Kristin Omsland, Katariina Laine, and Maria Christine Magnus in Women’s Health.

## Data Availability

Data can be requested from the Norwegian Mother, Father, and Child Cohort study (mobaadm@fhi.no) pending required ethical approval from The Regional Committee for Medical and Health Research Ethics in Norway.
