# Supplementary material for: Relationship and mental health outcomes after childbirth among women with endometriosis: An 8-year follow-up study
Source: Womens Health (Lond). 2026 Jul 22;22:17455057261471816. doi: 10.1177/17455057261471816 (PMC13392347; doi:10.1177/17455057261471816)
Supplement: Supplemental material - Relationship and mental health outcomes after childbirth among women with endometriosis: An 8-year follow-up studyRelationship and mental health outcomes after childbirth among women with endometriosis: An 8-year follow-up study [file sj-pdf-1-whe-10.1177_17455057261471816.pdf]

Supplementary Material:

RELATIONSHIP SATISFACTION SCALE

10-item scale (RSS-10)

1. I have a close relationship with my spouse/partner
2. My partner and I have problems in our relationship
3. I am very happy with our relationship
4. My partner is generally understanding
5. I often consider ending our relationship
6. I am satisfied with my relationship with my partner
7. We frequently disagree on important decisions
8. I have been lucky in my choice of a partner
9. We agree on how children should be raised
10. I think my partner is satisfied with our relationship

5-item scale (RSS-5)

2. My partner and I have problems in our relationship
3. I am very happy with our relationship
4. My partner is generally understanding
6. I am satisfied with my relationship with my partner
9. We agree on how children should be raised

(Questions 2, 3, 4, 6 and 9 from RSS-10)

Response options

1-Agree completely

2-Agree

3-Agree somewhat

4-Disagree somewhat

5-Disagree

6-Disagree completely

Score

Minimum score: 10, maximum score: 60.

A mean item score of 4.0 or higher serves as the cutoff for dissatisfaction with the relationship.

THE HOPKINS SYMPTOMS CHECKLIST

8-item scale (SCL-8)

Have you been bothered by any of the following during the last two weeks?

1. Feeling fearful (A)
2. Nervousness or shakiness inside (A)
3. Feeling hopeless about the future (D)
4. Felling blue (D)
5. Worrying too much about things (D)
6. Feeling everything is an effort (D)
7. Feeling tense or keyed up (A)
8. Suddenly scared for no reason (A)

Four items (1, 2, 7 & 8) assess anxiety (A) and four items (3, 4, 5 & 6) assess depression (D).

Response options

1-Not bothered

2-A little bothered

3-Quite bothered

4-Very bothered

Score

Minimum subscale scores (four items each): 4, maximum scores: 16.

An average score of ≥1.75 on a subscale indicates clinically significant symptoms of anxiety or depression.
