# Supplementary material for: Relationship and mental health outcomes after childbirth among women with endometriosis: An 8-year follow-up study
Source: Womens Health (Lond). 2026 Jul 22;22:17455057261471816. doi: 10.1177/17455057261471816 (PMC13392347; doi:10.1177/17455057261471816)
Supplement: Supplemental material - Relationship and mental health outcomes after childbirth among women with endometriosis: An 8-year follow-up studyRelationship and mental health outcomes after childbirth among women with endometriosis: An 8-year follow-up study [file sj-pdf-2-whe-10.1177_17455057261471816.pdf]

Supplementary Table 1. Relative risk (RR) of anxiety and depression for women with endometriosis (pregnancies with no reported endometriosis as reference) among 99,531 liveborn singleton pregnancies in the Norwegian Mother, Father and Child cohort study (1999-2008), with follow-up at 6 months, 18 months, and at 3, 5, and 8 years postpartum.

	Endometriosis n cases/n total (percentage)		No reported endometriosis n cases/n total (percentage)		Unadjusted RR		Adjusted RR[a]		Adjusted RR[b]	
**Anxiety**										
6 months postpartum	98/1,164	(8.42 %)	6,244/75,795	(8.24 %)	1.02	(0.84-1.24)	1.09	(0.90-1.32)	1.06	(0.87-1.29)
18 months postpartum	117/1,096	(10.68 %)	6,838/70,604	(9.68 %)	1.10	(0.93-1.31)	1.16	(0.97-1.38)	1.14	(0.96-1.35)
3 years postpartum	107/893	(11.98 %)	5,572/54,150	(10.29 %)	1.16	(0.97-1.40)	1.20	(1.00-1.45)	1.17	(0.97-1.41)
5 years postpartum	58/635	(9.13 %)	2,764/38,892	(7.11 %)	1.29	(1.00-1.65)	1.37	(1.07-1.77)	1.33	(1.03-1.71)
8 years postpartum	91/665	(13.68 %)	4,329/40,172	(10.78 %)	1.27	(1.04-1.55)	1.33	(1.09-1.62)	1.30	(1.06-1.58)
**Depression**										
6 months postpartum	212/1,164	(18.21 %)	12,454/75,733	(16.44 %)	1.11	(0.97-1.25)	1.15	(1.01-1.30)	1.14	(1.01-1.29)
18 months postpartum	243/1,090	(22.29 %)	14,820/70,536	(21.01 %)	1.06	(0.95-1.19)	1.10	(0.98-1.24)	1.09	(0.97-1.22)
3 years postpartum	222/893	(24.86 %)	11,077/54,077	(20.48 %)	1.21	(1.08-1.37)	1.25	(1.11-1.40)	1.23	(1.09-1.38)
5 years postpartum	106/635	(16.69 %)	5,676/38,801	(14.63 %)	1.14	(0.95-1.37)	1.20	(1.00-1.43)	1.17	(0.98-1.40)
8 years postpartum	184/668	(27.54 %)	8,455/40,209	(21.03 %)	1.31	(1.15-1.49)	1.35	(1.19-1.53)	1.32	(1.16-1.50)

[a]*adjusted for maternal age at birth*

[b]*adjusted for maternal age at birth and socioeconomic status (maternal education and income)*
